# Three species of *Echinococcus granulosus* sensu lato infect camels on the Arabian Peninsula

**DOI:** 10.1007/s00436-021-07156-1

**Published:** 2021-04-17

**Authors:** Fahad A. Al-Hizab, Nouh S. Mohamed, Marion Wassermann, Mahmoud A. Hamouda, Abdelazim M. Ibrahim, Waleed R. El-Ghareeb, Sherief M. Abdel-Raheem, Thomas Romig, Rihab A. Omer

**Affiliations:** 1 Department of Pathology, College of Veterinary Medicine, King Faisal University, Hofof, Saudi Arabia; 2 Department of Parasitology and Medical Entomology, Nile University, Khartoum, Sudan; 3 Parasitology Unit 190p, Institute of Biology, University of Hohenheim, Emil-Wolff-Straße 34, 70599 Stuttgart, Germany; 4 Department of Veterinary Public Health and Animal Husbandry (Meat Hygiene), College of Veterinary Medicine, King Faisal University, Hofof, Saudi Arabia; 5 Department of Molecular Biology, Institute of Parasitology, University of Leipzig, Leipzig, Germany

**Keywords:** *Echinococcus granulosus* sensu stricto, *E. ortleppi*, *E. canadensis* G6/7, Camels, Kingdom Saudi Arabia, Strains

## Abstract

We report on the genetic identity of 36 *Echinococcus* cysts that were collected during a recent slaughterhouse survey of 810 locally bred camels (dromedaries) in the Eastern Province of the Kingdom of Saudi Arabia. Analysis of a partial *nad1* gene sequence showed that the majority (*n* = 29) belonged to *E. granulosus* sensu stricto, four to *E. canadensis* G6/7, and three to *E. ortleppi*. Eight of the 29 *E. granulosus* s.s. cysts contained protoscoleces; all other cysts were calcified and non-viable. This is the first report of the presence *E. ortleppi* from the Arabian Peninsula, a parasite that is typically transmitted via cattle. The results indicate widespread infection of camels with CE in eastern Saudi Arabia and an active role of camels in the lifecycles of at least *E. granulosus* s.s.. Complete *cox1* haplotype analysis of 21 *E. granulosus* s.s. isolates shows that the majority of variants circulating in eastern Saudi Arabia is distinct from but closely related to haplotypes from neighboring countries in the Middle East, which indicates the presence of this parasite in KSA for a longer period of time. All isolates of *E. granulosus* s.s. in this study belonged to the G1 cluster, although the G3 genotype has previously also been reported from the Middle East.

## Introduction

Cystic echinococcosis (CE) is a zoonosis caused by cestodes of the species complex *Echinococcus granulosus* sensu lato (s.l.). The adult tapeworms are asymptomatic intestinal parasites of dogs and wild carnivores which get infected by ingesting cystic larval stages (metacestodes) that develop in herbivorous intermediate hosts (usually via offal after uncontrolled slaughter of livestock) (Eckert and Deplazes 2004; Moro et al. 2008). Cysts can grow in almost all organs of intermediate hosts, most often the liver and lungs, causing various degrees of morbidity and mortality depending on species of host and parasite, cyst size, and location. Apart from livestock (sheep, goats, cattle, camels, donkeys) and wild herbivores, humans can acquire CE by accidental ingestion of *Echinococcus* eggs from the environment (Eckert and Deplazes 2004).

To date, five species of *Echinococcus* are known to cause CE in animals or humans. *Echinococcus granulosus* sensu stricto (s.s.) is most frequently transmitted in a lifecycle involving domestic dogs and sheep, although a range of other mammals (including camels) are also suitable intermediate hosts; this parasite is responsible for the majority of human cases worldwide (Alvarez Rojas et al. 2014). *Echinococcus equinus* is adapted to equids (horses and donkeys) as intermediate hosts, *E. ortleppi* to cattle, and *E. canadensis* consists of genotypes that frequently infect camels, pigs, and wild herbivores. The fifth species, *E. felidis*, is a wildlife parasite of sub-Saharan Africa that may not be zoonotic. All these taxa show marked differences in host range and pathogenicity (also to humans), but are difficult to differentiate morphologically, particularly in the cyst stage. Molecular characterization of the causative agents of CE is therefore mandatory to gather information on transmission patterns and control options (Alvarez Rojas et al. 2014).

Few such data on CE are available from the Kingdom of Saudi Arabia (KSA), although the presence and frequency in humans and livestock are well documented. Substantial numbers of human CE cases were reported from KSA (Fahim and Al Salamah 2007; Al-Malki and Degheidy 2013), as well as from neighboring Yemen, Oman, and Qatar (Deplazes et al. 2017). In livestock, prevalences reported from KSA are 0.5–34.6% in camels, 0.0–28.7% in cattle, 0.1–69.6% in sheep, and 0.5–19.8% in goats. Most studies were done in the western and central parts of the country, except one study each on camels and sheep in Eastern Province (Farah 1987; Haroun et al. 2008; Ibrahim 2010; Toulah et al. 2012; Al-Malki and Degheidy 2013; Hayajneh et al. 2014; Almalki et al. 2017; El-Ghareeb et al. 2017; Toulah et al. 2017; Abdel-Baki et al. 2018; Fdaladdin et al. 2018; Al-Hizab et al. 2018).

The KSA is a major importer of livestock for slaughter and consumption from other Middle Eastern and African countries. Prevalence figures from slaughterhouse survey often do not discriminate between locally raised and imported animals. However, prevalence differences may be substantial: Al-Malki and Degheidy (2013) found CE in 9.6% of local, but 29.2% of imported sheep, while Fdaladdin et al. (2018) and El-Ghareeb et al. (2017) found local sheep slightly more frequently infected than imported sheep in Al-Madina and Al-Ahsa, respectively.

The molecular identification of the causative *Echinococcus* species in KSA is limited to two studies: Metwally et al. (2018) identified only *E. granulosus* s.s. among 18 fertile cysts from sheep and camels from Riyadh, and Al-Mutairi et al. (2020) identified one cyst each from sheep and camel from western KSA as *E. granulosus* s.s.. In neighboring Oman, *E. granulosus* s.s. was found in camels, cattle, sheep, and goats, and *E. canadensis* G6/7 in camels and cattle (Al-Kitani et al. 2015; Al-Kitani et al. 2020). One isolate from a human patient in Yemen was identified as *E. granulosus* s.s. (Alam-Eldin et al. 2015).

Here, we provide first insights into the causative agents of CE circulating in local camel breeds in the eastern parts of KSA. It is intended as a baseline study, to be followed by more comprehensive surveys of other livestock species in this vast and under-researched region.

## Material and methods

### Study area and origin of samples

The Eastern Province (asch-Sharqiyya), bordering Iraq, Kuwait, Qatar, UAE and Oman, is the largest province of the Kingdom of Saudi Arabia in terms of area and the third in terms of human population. More than half of the 673,000 km^2^ area is covered by the uninhabitable Rub´al Khali desert. No precise numbers of livestock are available for the province, but camels, sheep, and goats are the principle species kept.

The samples used in this study had been collected in the course of a survey by Al-Hizab et al. (2018), who found *Echinococcus* cysts in the livers of 216 out of 810 dromedaries (*Camelus dromedarius*) of different local breeds slaughtered for meat in the abattoirs of Al Omran, Al Ahsa, and Al Dammam; one cyst per camel was isolated for further investigation, while cysts in locations other than the liver were not recorded and collected. Age and sex of the examined animals were documented, as well as cyst size and fertility status. Metacestode tissue and cyst fluid were transferred into sterile tubes with 70% EtOH. Protoscoleces were detected in eight cysts. These, and further 59 randomly selected non-fertile cysts, each from different camels, were provided for molecular identification of the causative *Echinococcus* species.

### DNA preparation

DNA was prepared using the 0.02 M NaOH method as described previously (Hüttner et al. 2008). Briefly, cyst fluid was centrifuged and examined microscopically, and one protoscolex or a tissue piece of similar size was transferred in a volume of 1 μl cyst fluid via pipette to 10 μl of 0.02 M NaOH solution and heated up to 95 °C for 15 min. The resulting lysate was used directly as DNA template for the PCR.

### Polymerase chain reaction of partial *nad1* gene for species differentiation

For species identification, a 189 bp long fragment of the *nad1* gene was analyzed. The amplification was carried out using the forward and reverse primers 5′ TGT TTT TGA GAT CAG TTC GGT GTG ′3 and 5′ CAT AAT CAA ACG GAG TAC GAT TAG ′3, respectively. If necessary, a second/nested PCR was performed using the forward primer 5′ CAG TTC GGT GTG CTT TTG GGT CTG ′3 and the reverse primer 5′ GAG TAC GAT TAG TCT CAC ACA GCA ′3 (Hüttner et al. 2008). PCR reaction mixture was in total of 25 μl containing 10 pmol of either the first PCR or nested PCR primers, 10 mM Tris-HCl (pH 8.3), 50 mM KCl, 2 mM MgCl_2_, 200 μM of each dNTP, 0.125 U Ampli-*Taq* polymerase (Applied Biosystems), and 2 μl of the lysate in the first PCR or 2 μl first-PCR-product as a template for the second PCR. PCR amplification conditions were identical for both PCRs as follows: an initial denaturation step at 95 °C for 5 min followed by 35 cycles of denaturation at 95 °C for 30 s, annealing at 55 °C for 30 s, and extension at 72 °C for 30 s. After a final extension step at 72 °C for 5 min, the PCR products were cooled down to 4 °C.

### Polymerase chain reaction of the *cox1* gene

For further phylogenetic and haplotype analyses of the *E. granulosus* s.s. samples, the complete *cox1* gene was amplified and sequenced. PCR was performed using primers described previously (Hüttner et al. 2008). In brief, for the first PCR a 25 μl reaction containing 10 pmol of the forward and the reverse outer primers (*cox1* outer forward primer: 5′ TTA CTG CTA ATA ATT TTG TGT CAT ′3 and *cox1* outer reverse primer: 5′ GCA TGA TGC AAA AGG CAA ATA AAC ′3), 10 mM Tris-HCl (pH 8.3), 50 mM KCl, 2 mM MgCl_2_, 200 μM of each dNTP, 0.125 U Ampli-*Taq* polymerase (Applied Biosystems), and 2 μl of the lysate. The amplicons produced by the first primers were used as DNA templates for the nested PCR using the nested primer pair (*cox1* nested forward primer: 5′ GTT AGT TTT GAC TGT ACG TTT TCA ′3 and *cox1* nested reverse primer: 5′ GAC TAA TAA TCA ACT TAG ACT TAC ′3). Reaction mixture was set up to 50 μl using 20 pmol of each nested primer, 10 mM Tris-HCl (pH 8.3), 50 mM KCl, 2 mM MgCl_2_, 200 μM of each dNTP, 0.25 U Taq polymerase, and 2 μl of the PCR amplicons. Conditions of amplifications were identical for both PCRs. PCR conditions were as follows: an initial denaturation step at 95 °C for 5 min followed by 35 cycles of denaturation at 95 °C for 30 s, annealing at 55 °C for 30 s, and extension at 72 °C for 2 min. After a final extension step at 72 °C for 5 min, the PCR products were cooled down to 4 °C.

### PCR product visualization and sequencing

PCR products were visualized using 1.5% agarose gel (GENAXXON) stained with GelRed (Biotum). Five microliter of each PCR product were mixed with 1 μl 6 × DNA loading dye (Thermo scientific) and loaded on the gel. Electrophoresis was carried out at 5 V/cm for 20 min. PCR products were viewed under UV light transilluminator (INTAS). The obtained amplicons were purified using the High Pure PCR Product Purification Kit (Roche) following the manufacturers instruction. PCR amplicons’ lengths were compared against a middle range DNA ladder (Thermo scientific).

PCR amplicons were sequenced by GATC-biotech company (Germany).

### Genetic diversity analysis

*Echinococcus* species identification of amplified *nad1* gene was done by comparison with available sequences in the NCBI GenBank database using the BLAST nucleotide algorithm (http://www.ncbi.nlm.nih.gov/Blast.cgi).

Obtained chromatograms of the *E. granulosus* s.s. *cox1* sequences were viewed and manually edited using GENtle v. 1.9 [University of Cologne, Germany (Manske 2006)] and aligned by MEGA7 (Kumar et al. 2016). Indices of population diversity such as the number of haplotypes (H), segregating sites (S), haplotypes diversity (Hd) and the average number of nucleotide differences between two sequences (p); Tajima’s D test, Fu and Li’s D* and F* statistics analysis were obtained using DnaSP v5.10 to estimate the neutral theory of natural selection. For the construction of the phylogenetic tree and haplotype analysis, the complete *cox1* sequences of previously published *E. granulosus* s.s. sequences from different geographic regions are used as reference sequences (Table 1). The sequences were selected based on their origin, focusing on isolates from countries in the Middle East known to export livestock to Saudi Arabia. In addition to these, sequences were included that most closely matched the haplotypes found in the present study after BLAST search. The construction of the phylogenetic tree was based on the maximum likelihood method. The model with the lowest BIC scores (Bayesian information criterion) was considered the best model to describe the nucleotides substitution patterns. Tamura-Nei’s model was used for constructing the phylogenetic tree with 1000 bootstraps using MEGA7 software. *Taenia solium* was used as outgroup taxon [AB066493.1; (Nakao et al. 2002)]. The parsimony haplotype networks were calculated using the online TCS software v 1.8 (Clemet et al. 2000) and tcsBU (Múrias dos Santos et al. 2016).

**Table 1:** Origin and accession numbers of *cox1* sequences used for phylogenetic analyses

Region	Accession no.	Reference	Region	Accession no.	Reference
North Africa			Western Asia		
Tunisia	MG672267 MG672268 MG672269 MG672271 MG672273 MG672276	(Kinkar et al. 2018a)	Jordan	AB688594 AB688599 AB688601	(Yanagida et al. 2012)
			Iran	JQ250807 JQ250810 JQ250813	
Algeria	MG808312 MG808345 MG808346 MG808348	(Laatamna et al. 2019)		MG682537 MG682538 MG682539 MG682540 MG682541	(Kinkar et al. 2018b)
**South America**				MG672248 MG672244 MG672238	(Kinkar et al. 2016)
Argentina	KX039947	(Laurimae et al., 2016)			
	MG672210 MG672214 MG672258	(Kinkar et al. 2018a)			
				GQ856692	(Nejad et al. 2012)
				JQ219963	Mohammadzadeh et al., Unpublished
Brazil	MG672224 MG672226 MG672229		Turkey	KU925378 KU925404 KU925358	(Kinkar et al. 2016)
Peru	AB688620	(Yanagida et al. 2012)		MG682530 MG682531 MG682534 MG682536	(Kinkar et al. 2018b)
**Europe**					
France	MG682520	(Kinkar et al. 2018b)			
Italy	MG682522				
	MG672278 MG672133 MG672281	(Kinkar et al. 2018a)	Armenia	KX020342 KX020377 KX020403	Ebi et al., unpublished
Finland	MG672132		**Far East Asia**		
Moldova	MG672145 MG672146		Russia	AB777905 AB777906 AB777907 AB777908	(Konyaev et al. 2013)
Romania	MG672131				
Spain	MG672137				
	KU925419	(Kinkar et al. 2016)	China	AF297617	(Le et al., 2002)
**South Asia**			**Australia**		
India	MG682542 MG682543	(Kinkar et al. 2018b)	Australia	NC044548	(Kinkar et al. 2019)
				KT968705	(Alvarez Rojas et al. 2016)

## Results

### Species identification of *Echinococcus* spp.

Species differentiation using a small fragment of the *nad1* gene was successful with 36 out of 67 cysts. *Echinococcus granulosus* s.s. was dominant among the analyzed samples (29/36), followed by *E. canadensis* (4/36) and *E. ortleppi* (3 / 36). All cysts were located in the liver (other organs were not examined) and measured less than 4 cm in diameter. Data on fertility status of the cysts as well as age and sex of the camels are presented in Table 2.

**Table 2:** Age and sex of the camels and fertility status of the cysts that could be sequenced

Category			
	*E. granulosus* s.s.	*E. ortleppi*	*E. canadensis*
Age of camel			
< 5 years	7	0	0
5–10 years	9	1	1
> 10 years	13	2	3
Sex of camels			
Male	11	1	2
Female	18	2	2
Cyst status			
Fertile	8	0	0
Sterile	0	0	0
Calcified	21	3	4
Total	29	3	4

### Genetic diversity and phylogenetic analyses

The complete *cox1* gene could be successfully amplified from 21 out of the 29 *E. granulosus* s.s. isolates. Nine different haplotypes were found, six of which had not been described before. Sequences were deposited in GenBank under the accession numbers MW051119- MW051127.

Comparison with GenBank entries revealed that KSA haplotypes were most closely related with sequences found in western Asia including the Middle East (Table 3). The haplotype H06 from our panel is identical with the most common genetic variant of *E. granulosus* s.s. which can be found worldwide.

**Table 3:** Similarity with deposited sequences in the GenBank database

Haplotype	No. and names of KSA isolates	Similarity with closest sequences	Origin of closest sequences
H01	*n* = 7 (KSA-1, 3, 5, 12, 13, 14, 16)	99.94 %	Armenia, Jordan
H02	*n* = 3 (KSA-17, 19, 20)	100 %	Iran, Turkey, Russia
H03	*n* = 2 (KSA-2, 9)	99.88 %	Iran, Moldova
H04	*n* = 2 KSA-4, 6)	99.81 %	Algeria, Armenia, Jordan
H05	*n* = 2 (KSA-11, 15)	99.88 %	Armenia, Jordan
H06	*n* = 2 (KSA-18, 21)	100 %	global distribution
H07	*n* = 1 (KSA-7)	100 %	Iran
H08	*n* = 1 (KSA-8)	99.94 %	Iran
H09	*n* = 1 (KSA-10)	99.88 %	Algeria, Armenia, Jordan

**Fig. 1:**
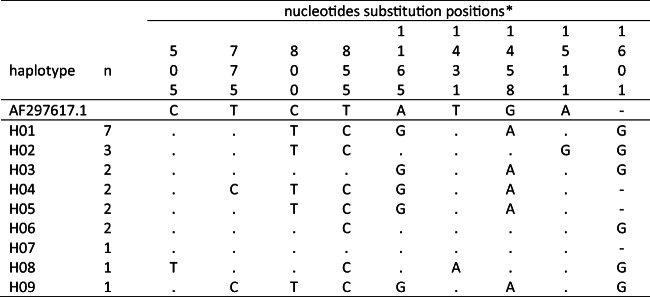
Sequence alignment of the 9 haplotypes. Substitutions were indicated with their nucleotide code, deletions were marked by ( - ), and dots ( . ) indicate identical nucleotide at the specified position in comparison with the reference sequence AF297617 (Lee et al. 2002). *nucleotides substitution positions, based on the start of the complete *cox1* gene, read vertically

**Fig. 2:**
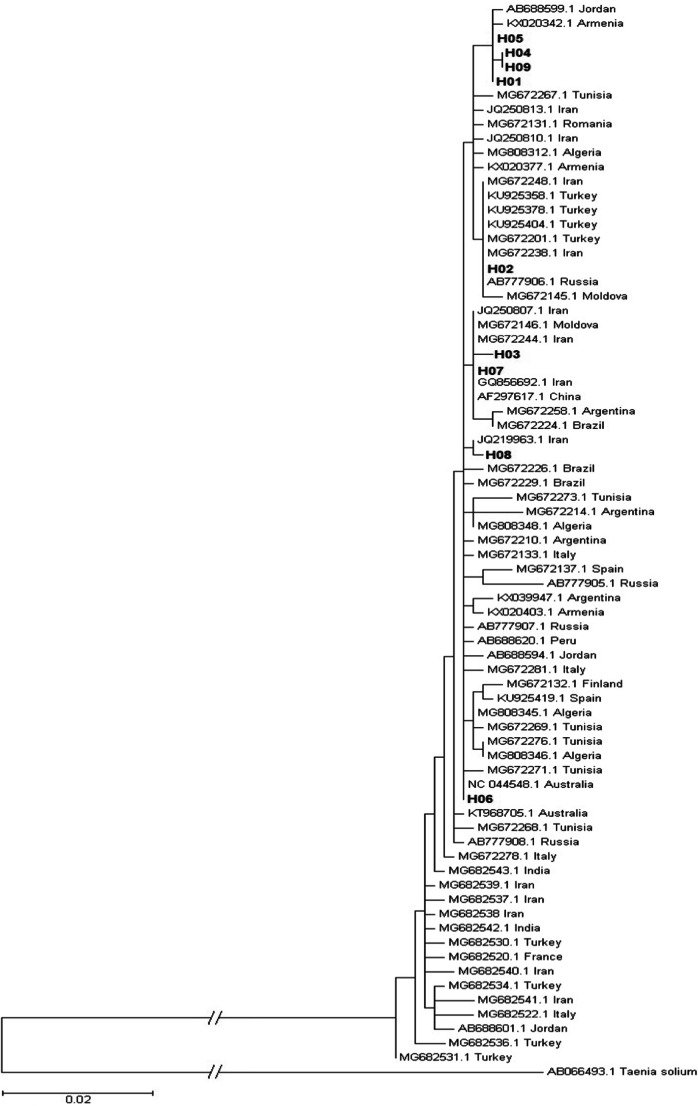
Phylogenetic tree showing the relation between the Saudi Arabian haplotypes with 65 reference sequences. The Saudi Arabian haplotypes (H01-09) are in bold. The reference sequences along with their accession numbers and origin of isolate were included for each. *T. solium* was used as an outgroup taxon. The branch to outgroup was shortened by 0.2 substitutions per site

The nine haplotypes from Saudi Arabia differ within the *cox1* gene at nine positions. The exchanges or insertions with respect to the reference sequence AF297617 (Lee et al. 2002) are shown in Fig. 2. Seven of the sites are parsimony-informative (Fig. 1).

A phylogenetic tree was constructed to investigate the relation of the 21 camel samples with 65 previously published sequences from different geographic regions. Four of the Saudi Arabian haplotypes, representing 12 isolates, cluster close together in the topmost clade; the other five haplotypes are dispersed over the tree (Fig. 2).

**Fig. 3:**
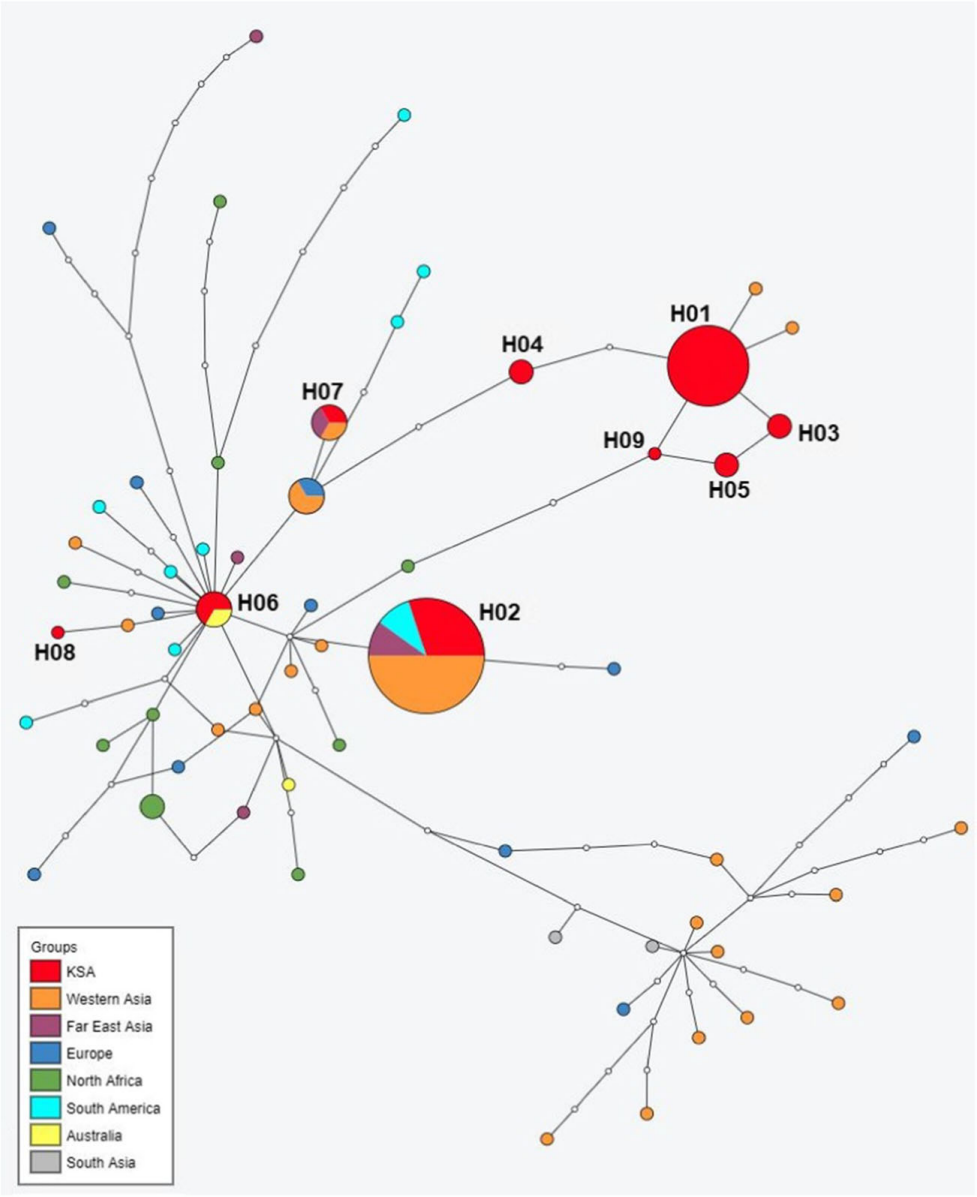
Parsimony haplotype network of the 21 camel isolates and 65 worldwide selected sequences. Haplotypes of each region are presented using color coding. White nodes represent hypothetical haplotypes. Sizes of colored nodes are proportional to the number of isolates found per haplotype. The cluster on the lower right side (without KSA isolates) is formed of sequences conforming to the G3 genotype

The resulting haplotype network shows two main clusters (Fig. 3): one centered on the most common globally distributed haplotype, which was represented by only two isolates in our sample set (H06). The second cluster, conforming to the genotypic G3 cluster, is formed by Asian and European haplotypes without any representatives from KSA. Five of the nine KSA haplotypes (representing 14 of 21 isolates) are closely related to each other and not known from other areas.

The analysis of the neutrality indices showed high haplotype diversity (Hd) of 0.790 ± 0.00548 with low nucleotide diversity (Pi) of 0.00145, and the number of polymorphic (segregating) sites (S) detected was 8. The high Hd, low Pi, and the small number of S all indicate high diversity within the isolates. The average number of pairwise nucleotide differences (*k*) was 2.324. Fu and Li’s D* test statistic value was 0.19214 (*P* > 0.10); Fu and Li’s F* test statistic value was 0.20851 (*P* > 0.10). Testing the natural selection theory resulted in a Tajima’s D value of 0.14980 (*P* > 0.10). The calculated Fu and Li’s D* and F* tests and Tajima’s D test showing positively statistically insignificant values indicate that the population had undergone expansion.

## Discussion

Only two previous studies had addressed the frequency of CE in the eastern region of Saudi Arabia: 26.7% of 810 locally raised camels were infected, based on a slaughterhouse survey in Al-Dammam, Al-Omran, and Al-Ahsa slaughterhouses (Al-Hizab et al. 2018); however, this is likely to be a gross underestimate as only livers were examined for CE, and most cysts in camels appear to occur in the lungs (Omer et al. 2010; SHAHNAZI et al. 2011). In addition, 563 native sheep were examined in Al-Ahsa, of which 7.7% were infected (El-Ghareeb et al. 2017). These figures range on a similar level as those from other regions of Saudi Arabia. Other countries on the Arabian Peninsula are data deficient, except for Oman where CE was found in all species of livestock albeit at low prevalence: a slaughterhouse survey conducted 2010–2013 found prevalences of 5.3% in camels, 0.6% in cattle, and < 1% in sheep and goats (Al-Kitani et al. 2015). From neighboring Kuwait, only older data exist, where CE was found in 39.6% of 293 slaughtered camels in the 1980s (Abdul-Salam and Farah 1988).

Based on molecular diagnosis, only *E. granulosus* s.s. had previously been found in KSA among 20 isolates from sheep and camels (Metwally et al. 2018; Al-Mutairi et al. 2020). The predominance of this species in KSA seems to be confirmed by our results, as the majority of cysts in our sample (29 of 36) belonged to *E. granulosus* s.s., only four and three to *E. canadensis* and *E. ortleppi*, respectively. However, this frequency distribution is unlikely to reflect the true situation, as *E. canadensis* and *E. ortleppi* are most often found in the lungs of their respective hosts (Omer et al. 2010; Banda et al. 2020), which were missed in this study as lung cysts had not been collected. Likewise, the fact that all cysts of *E. canadensis* and *E. ortleppi* were calcified in our sample is likely due to this sampling bias and does neither reflect the frequency of these parasite species nor the role of camels in their transmission. Camels are highly competent hosts for *E. canadensis* G6/7, formerly named the “camel strain”, which is the most frequent *Echinococcus* taxon in camels worldwide (Bold et al. 2019; Dehghani et al. 2020; Anvari et al. 2021). In a major survey in Sudan, 74% of 2378 *E. canadensis* G6/7 cysts in camels were fertile (Omer et al. 2010). There are only four additional published records of camel infection with *E. ortleppi*, from Sudan, Egypt, and Iran, and in at least two of them, fertile cysts were described (Ahmed et al. 2013; Amer et al. 2015; Aziz and El Meghanawy 2016; Ebrahimipour et al. 2017); this parasite mainly affects cattle (Romig et al. 2017) but may use additional host species for transmission in certain areas (e.g., domestic pigs in Zambia (Banda et al. 2020)). Therefore, the role of camels in the lifecycle of *E. ortleppi* remains to be investigated.

All isolates for our studies were collected from local camel breeds, which means that three different species of *E. granulosus* s.l. circulate in the eastern region of KSA. Additional studies are needed to address the quantitative contribution of different species of livestock to their transmission. Very little data are available on the epidemiology of CE in the KSA apart from prevalence data from livestock; domestic dogs are highly likely to be the primary definitive hosts for all *Echinococcus* spp. in KSA, but the circumstances of transmission are unclear. Unsafe disposal of offal in slaughterhouses are major infection sources for dogs in many countries, but the economic standards of KSA have facilitated state-of-the-art slaughtering facilities (Dar and Alkarmi 1997). There is only one older study that reported *Echinococcus* infection in dogs in KSA (15.6% prevalence in Al-Ahsa region, Eastern Province) (Kawasmeh et al. 1984).

The Kingdom of Saudi Arabia is known for large-scale import of livestock from other Middle Eastern and African countries (e.g., Sudan). In most of these countries, CE is highly prevalent; therefore, the chance of importing infected animals is high. Some of the above cited slaughterhouse surveys for CE do not discriminate between local and foreign animals and those that do give no information on the origin of imported animals. It is therefore not surprising that in some studies, local breeds are more frequently infected than imported animals, and vice versa in other studies (Al-Malki and Degheidy 2013; Toulah et al. 2017; El-Ghareeb et al. 2017; Fdaladdin et al. 2018). In any case, it is of concern if such large-scale importation of parasites together with livestock results in “escapes” of these parasites into local circulation. This is particularly important in case of *E. granulosus* s.s., which is the causative organism of by far the largest proportion of human CE cases worldwide (Alvarez Rojas et al. 2014). We therefore compared the *cox1* haplotypes from our *E. granulosus* s.s. isolates with haplotypes available from other countries in the Middle Eastern region and worldwide. Interestingly, most haplotypes found in our panel (6 of 9) were unique and had not been reported from outside KSA but were most closely related with haplotypes that are known from Iran or Jordan. The three haplotypes that had been described before (H02, H06, and H07) are all known from the Middle Eastern region as well (Iran, Jordan, Turkey); it is noteworthy that H06, the most common and most widely distributed variant worldwide, is comparatively rare in our panel of samples. This genetic structure with their high “endemicity rate”, high haplotype diversity, close relatedness (low nucleotide diversity), and the rarity of the ubiquitous H06 (with only two isolates) leads to the hypothesis that *E. granulosus* s.s. is of autochthonous presence at least in this part of the Arabian Peninsula and does not depend on importation. This hypothesis needs to be tested with a larger set of isolates, including samples from livestock species other than camels.

Further genetic analyses are also warranted concerning the origin of *E. ortleppi* and *E. canadensis* in KSA. Although infection of local camels in this study indicates local transmission, it cannot be excluded that these parasites had been introduced from elsewhere. As examples, *E. ortleppi* is highly prevalent in cattle in Brazil (Monteiro et al. 2016), and *E. canadensis* G6/7 is the predominant species in Sudan (Omer et al. 2010), the two countries which account for the vast majority of imported livestock into KSA (WITS 2015).

In summary, CE of livestock in KSA is widespread, largely autochthonous at least for *E. granulosus* s.s.; camels are an important component of their lifecycles, but the precise background of transmission and possible origin of the parasites is in need of investigation.
